# Fractionated radiosurgery for painful spinal metastases: DOSIS - a phase II trial

**DOI:** 10.1186/1471-2407-12-530

**Published:** 2012-11-19

**Authors:** Matthias Guckenberger, Maria Hawkins, Michael Flentje, Reinhart A Sweeney

**Affiliations:** 1 Department of Radiation Oncology, University of Würzburg, Josef-Schneider-Str. 11, Würzburg, 97080, Germany; 2 The Royal Marsden Hospital NHS Foundation Trust, London, United Kingdom

**Keywords:** Phase II trial, Spinal metastasis, Pain, Radiosurgery, Stereotactic body radiotherapy

## Abstract

**Background:**

One third of all cancer patients will develop bone metastases and the vertebral column is involved in approximately 70% of these patients. Conventional radiotherapy with of 1–10 fractions and total doses of 8-30 Gy is the current standard for painful vertebral metastases; however, the median pain response is short with 3–6 months and local tumor control is limited with these rather low irradiation doses. Recent advances in radiotherapy technology – intensity modulated radiotherapy for generation of highly conformal dose distributions and image-guidance for precise treatment delivery – have made dose-escalated radiosurgery of spinal metastases possible and early results of pain and local tumor control are promising. The current study will investigate efficacy and safety of radiosurgery for painful vertebral metastases and three characteristics will distinguish this study. 1) A prognostic score for overall survival will be used for selection of patients with longer life expectancy to allow for analysis of long-term efficacy and safety. 2) Fractionated radiosurgery will be performed with the number of treatment fractions adjusted to either good (10 fractions) or intermediate (5 fractions) life expectancy. Fractionation will allow inclusion of tumors immediately abutting the spinal cord due to higher biological effective doses at the tumor - spinal cord interface compared to single fraction treatment. 3) Dose intensification will be performed in the involved parts of the vertebrae only, while uninvolved parts are treated with conventional doses using the simultaneous integrated boost concept.

**Methods / Design:**

It is the study hypothesis that hypo-fractionated image-guided radiosurgery significantly improves pain relief compared to historic data of conventionally fractionated radiotherapy. Primary endpoint is pain response 3 months after radiosurgery, which is defined as pain reduction of ≥ 2 points at the treated vertebral site on the 0 to 10 Visual Analogue Scale. 60 patients will be included into this two-centre phase II trial.

**Conclusions:**

Results of this study will refine the methods of patient selection, target volume definition, treatment planning and delivery as well as quality assurance for radiosurgery. It is the intention of this study to form the basis for a future randomized controlled trial comparing conventional radiotherapy with fractionated radiosurgery for palliation of painful vertebral metastases.

**Trial registration:**

ClinicalTrials.gov Identifier: NCT01594892

## Background

### Conventional palliative radiotherapy for painful vertebral metastases

Approximately one third of all patients with cancer will develop bone metastases [1] and of these patients, approximately 70% will have metastases involving the vertebral column, most commonly the thoracic and lumbar spine. Radiation therapy plays an important role in the multidisciplinary treatment of symptomatic vertebral metastases, including palliation of pain, control or prevention of neurological symptoms, and prevention of pathologic fractures.

During the past three decades, the gold standard of radiotherapy for painful bony metastases has been based on several randomized trials comparing various radiotherapy fractionation schemas: the majority of the studies compared single fraction radiotherapy of 8 Gy with fractionated protocols of 5–10 fractions and total doses of 20-30 Gy (meta-analyses in [2,3]). Radiotherapy was effective with overall pain relief in 70% of the patients on average. No dose–response relationship has been demonstrated in the meta-analyses meaning that increased irradiation doses delivered in multiple fractions did not result in higher pain response rates than a single fraction of 8 Gy and this palliative regime is consequently the evidence-based standard of care [4].

Nevertheless, there are clinical data suggesting that patients with painful vertebral metastases might benefit from higher irradiation doses than used in the prospective trials cited above. Despite overall pain response being high at 70%, the median duration of pain response is only 3 – 6 months. Consequently, only approximately 1/3 of the patients are effectively palliated for a duration of a few months with these conventional radiotherapy schemas. Additionally, partial pain response should not be the primary goal of treatment but rather complete pain response, which is achieved in only one quarter of the patients.

Studies on metastatic spinal cord compression (MSCC) confirm that conventional irradiation doses might not be sufficient for intermediate or even long term local disease control. Patchell et al. reported a randomized trial comparing radiotherapy alone with decompressive surgery followed by radiotherapy [5]; radiotherapy consisted of ten fractions of 3 Gy. Maintenance of walking ability was achieved for a median duration of 13 days and 122 days in the radiotherapy alone arm compared to combined treatment indicating that intensification of local treatment improved the outcome. Another randomized study compared lower and higher dose radiotherapy for MSCC [6]. Whereas no difference in response rates was observed for the total patient population, significantly improved response after high dose compared to low dose irradiation was observed in the subgroup of patients with unfavourable histologies (lung, kidney, gastrointestinal, head and neck, melanoma and sarcoma); a dose response relationship was not observed in patients with favourable histologies (lymphoma, seminoma, breast, prostate, myeloma). This again indicates that subgroups of patients might benefit from intensified local radiotherapy in this palliative setting.

Diagnostic possibilities and systemic treatments have significantly evolved in the recent years, with many patients surviving a metastatic state for years, which increases the need for more effective treatment of spinal metastases. Furthermore, prognostic scoring systems are available, which allow selection of patients with favourable traits [5-7]. These patients with life expectancy longer than 3 – 6 months might benefit from or even require a radiotherapy treatment offering more durable pain control, maintenance of quality-of-life and of neurological function.

### Radiosurgery for painful vertebral metastases

Stereotactic radiosurgery for intracranial metastases delivered either with or without whole brain radiation therapy, has been associated with very high rates of local tumour control, in the range of 85-95% across studies [7-12]. This stereotactic radiosurgery not only improved local tumor control but also overall survival when practiced in patients with a solitary brain metastasis. These results, in conjunction with very low complication rates related to the highly conformal nature of the treatment, have made intracranial radiosurgery an increasingly popular and available treatment modality.

Irradiation with such escalated “radiosurgical” doses has not been possible for spinal metastases because of the anatomical situation, where the tumor is very close to the spinal cord and frequently even wrapped around this critical organ-at-risk. Traditional technologies made it impossible to achieve a sufficiently high irradiation dose in this complex shaped target volume while simultaneously keeping the dose to the spinal cord within accepted tolerances [13]. Two recent advances in radiotherapy technologies have made it possible to transfer the radiosurgical concept from the brain to the vertebral region. Intensity-modulated radiotherapy (IMRT) allows the generation of convexly shaped dose distributions sparing the spinal cord from high irradiation doses. Image-guided (IGRT) verification of patient set-up enables accurate delivery of the planned dose distributions, which is especially important from a safety perspective because of the steep dose gradients between the target and the spinal cord.

In a recent survey, more than 40% of all radiation oncologists in the US stated that they practice spine radiosurgery and most institutions favour single-fraction radiosurgical techniques due to best patient comfort with a “one-shot” outpatient treatment in the context of limited life expectancy [14]. Rapid adoption of spinal radiosurgery is observed despite the paucity of prospective trials. Only one prospective study has been published so far [15] and the randomized RTOG 0631 is still accruing patients. Nevertheless, results of these radiosurgical studies are promising: local tumor control / pain control has been reported in 80-90% of the patients and this was achieved with low rates of severe toxicity [15-20]. The most serious toxicity – radiation induced myelopathy – has been reported in less than 1% of the patients in a large analysis of > 1000 treatments [21].

The concept of radiosurgery practiced as single fraction, however, has limitations. Due to the immediate proximity of vertebral metastases to the spinal cord, the treatment dose deliverable is limited by the tolerance of the spinal cord [21-24]. In cases of radiosurgical treatment of these spinal cord abutting tumors, the epidural tumor component has been identified as the most frequent site of treatment failure: both the spinal cord and adjacent epidural tumour involvement were spared from high biological doses of radiosurgery [16-18,25,26]. Consequently, many study protocols exclude tumors within a distance of < 3 mm to the spinal cord, as does the RTOG 0631 trial. This however precludes exactly those patients which are at the highest risk of cord compression.

### Rationale for this trial

The rationale for this trial is based on the same hypothesis as the single-fraction radiosurgical studies: to improve pain control and local tumor control via dose intensified radiotherapy for vertebral metastases. This trial will differ in three important aspects from the currently available literature and trials:

1) A prognostic score for overall survival (modified Mizumoto Score) will be used for selection of patients with favourable life expectancy [27], who are expected to benefit most from the intensified radiotherapy. The number for treatment fractions will be adjusted based on the modified Mizumoto Score to balance intensity and length of treatment with life expectancy. Selection of patients with long life expectancy will also allow for evaluation of late toxicity and long-term efficacy.

2) Hypo-fractionated radiosurgery with 5 and 10 treatment fractions will be practiced in this study. Based on radiobiological modelling, hypo-fractionated radiosurgery allows higher biologically effective doses to the tumor directly adjacent to the spinal cord than single-fraction protocols. Recurrences at the interface between the spinal cord and the epidural tumor are expected to be reduced by fractionation. This will expand the indications for dose intensified radiotherapy to tumors immediately abutting the spinal cord.

3) Failures in untreated parts of the vertebrae shall be avoided by the use of a simultaneous integrated boost concept. Similar to the single-fraction radiosurgical studies, dose intensification will be performed in the macroscopic tumour using dedicated CT and MR imaging. However, the non-involved parts of the vertebra will be treated with a second dose level (conventional dose) to avoid recurrences in the untreated parts of the vertebrae.

Preliminary results from the University Hospital Wuerzburg with dose intensified irradiation using 20 fractions of 3 Gy [28] are promising and support the hypothesis of this trial; Patients with long life expectancy were selected for fractionated radiosurgery resulting in an actuarial local control rate and overall survival of 88% and 63% after 2 years [13]. No acute or chronic toxicity > Grade 2 was seen.

## Methods/design of the trial

### Study design

**Figure 1:**
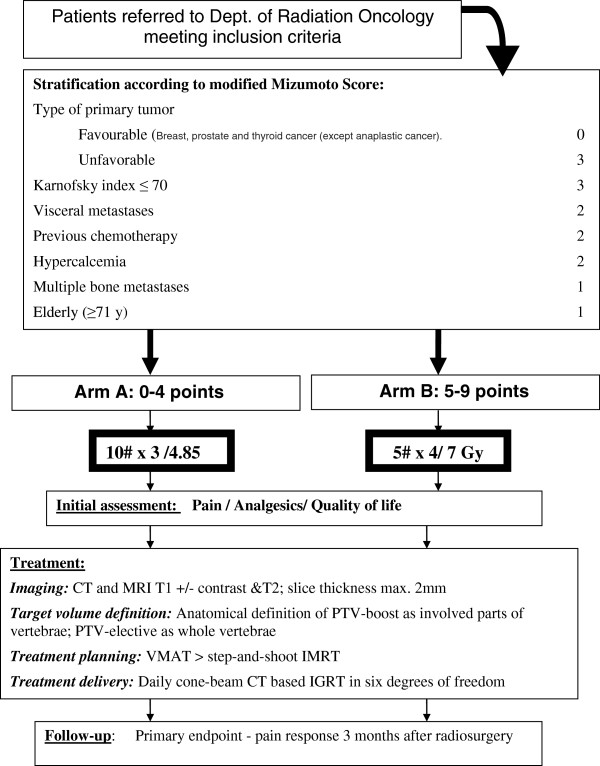
Design of the DOSIS study.

The study is designed as a prospective phase II trial. The outline of the study protocol is shown in Figure 1.

### Study hypothesis

Hypo-fractionated image guided radiosurgery significantly improves pain relief compared to historic data of conventionally fractionated radiotherapy.

### Primary endpoint

The primary objective is to determine whether a sustained pain relief can be achieved with dose intensified hypo-fractionated image-guided radiosurgery in patients with vertebral metastasis and intermediate and long life expectancy based on the modified Mizumoto Score. The Mizumoto score is slightly modified to improve overall survival in the group with intermediate life expectancy. Pain response 3 months after radiosurgery will be evaluated as primary endpoint and pain reduction of ≥ 2 points at the treated vertebral site on the 0 to 10 Visual Analogue Scale without analgesic increase will be defined as pain response [29].

### Secondary endpoints

1. Local tumor control at the treated vertebral levels and regional tumor control at the neighbouring vertebrae

2. Overall survival and cancer specific mortality

3. Quality of life using the EQ-5D and EORTC QLQ-BM22

4. Acute and late toxicity according to NCI CTCAE v 4.0

### Exploratory endpoints

1. Time between initial consult and first treatment (the goal is to reduce this period to within 48 hours by maximizing use of standardized procedures of diagnostics, fixation and planning)

2. Repositioning and image-guidance accuracy within the fixation system in six degrees of freedom

3. Intra-fraction motion as analysed by comparison of cone-beam CT imaging immediately preceding treatment

4. Morphological pattern of CT and MRI radiological response of vertebral metastases after dose-intensified, hypo-fractionated radiosurgery

### Inclusion criteria

Patients with the following characteristics will be eligible for this study

1. Established histological diagnosis of a malignant tumour (primary or metastatic)

2. Vertebral metastasis confirmed via biopsy or radiology

3. Pain in the involved spinal region or free of pain under pain medication

4. Fully consenting patients, > 18 years old

5. Karnofsky Performance Index ≥ 60%

6. Good or intermediate life expectancy according to the modified prognostic Mizumoto Score (score ≤ 9)

7. Patient must be able to tolerate fixation systems and 30 minutes treatment time

8. Discussed in interdisciplinary tumour board

9. The following types of spinal tumours are eligible:

Recurrent / residual tumours after surgery

Tumours in medically inoperable patients or patients deemed inoperable due to limited life expectancy / tumour load

Lesions associated with significant surgical risk

### Exclusion criteria

Patients with the following characteristics will be ineligible for this study

1. Short life expectancy according to the modified Mizumoto Sore

2. “Radiosensitive” histologies (i.e. lymphoma, SCLC, multiple myeloma)

3. Non-ambulatory status

4. Progressive neurological symptoms/deficit

5. > 3 involved vertebral levels

6. > 2 treatment sites

7. Spine instability

8. Previous radiotherapy at the involved levels

### Treatment planning

#### Patient positioning and imaging

No specific pre-medication will be applied. The patient will be placed in a stable position in the immobilization device (such as Body Fix). For cervical spine lesions, a combination of BodyFix and thermoplastic mask will be used. All patients will undergo a treatment planning CT scan immobilized in the treatment position with reconstruction of 2 mm axial slices. 3D volume imaging will be used for MRI imaging with 0.8-2 mm axial slices. T1 pre- and post-contrast as well as T2 sequences are required. The respective MRI scans will be registered with the treatment planning CT scan using rigid image registration.

#### Target and organs-at-risk delineation

**Figure 2:**
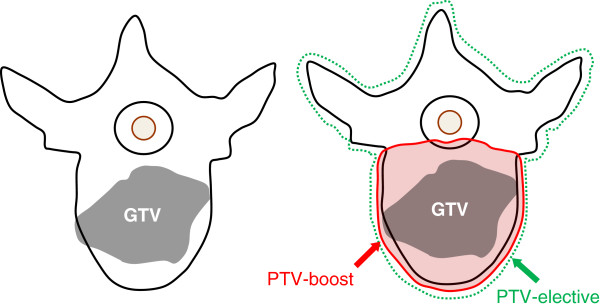
**Concept for definition of the PTV**-**boost and PTV**-**elective and illustration based on three cases with different GTV locations and macroscopic tumor extensions.**

The target volume concept is illustrated in Figure 2.

Gross tumour volume (GTV): The target lesion will be outlined on the planning MRI (GTV-MRI) and in cases without MRI imaging on the treatment planning CT scan (GTV-CT).

Planning target volume boost (PTV-boost): The PTV-boost will be based on an anatomical target volume concept, where all macroscopically involved elements of the involved vertebra (body, pedicles, transverse process, spinous process) are defined as PTV-boost.

PTV-elective: Defined as the entire vertebrae of the involved levels.

Organs at risk: The spinal cord will be contoured according to the MRI. A 1 mm expansion of the spinal cord will be performed to account for set-up errors, this being the spinal cord OAR. Additional organs-at-risk to be delineated are pharynx, oesophagus, lungs, kidneys and bowel.

#### Treatment planning

Patients will be treated with a linear accelerator with beam energies varying form 6-18MV. Inversely optimized intensity-modulated radiotherapy will be mandatory. Volumetric modulated arc therapy (VMAT) will be preferably utilized compared to step and shoot IMRT to minimize treatment times.

Two different fractionations will be used depending on estimated life expectancy: patients with long life expectancy (Arm A: Mizumoto score 0–4) will be treated with 10 fractions whereas patients with intermediate life expectancy (Arm B: Mizumoto score 5–9) will be treated with only 5 fractions. Total treatment doses will be 30 Gy and 48.5 Gy at the “PTV-elective” and at the “PTV-boost” for Arm A, respectively. Total treatment doses will be 20 Gy and 35 Gy at the “PTV-elective” and at the “PTV-boost” for Arm B, respectively. Two-Gray equivalent doses (α/β = 10 Gy) at the PTV-boost are 50 Gy and 60 Gy for the 5-fractions and 10-fractions regimen, respectively. The patients will be treated on consecutive workdays, with one fraction per day.

Normal tissue dose constraints are summarized in Table 1.

**Table 1:** **Organ**-**at**-**risk dose constraints**

	**Maximum dose**			**Mean Dose**	
	**Arm B**	**Arm A**	**Volume of max dose**	**Arm B**	**Arm A**
Fractionation Scheme	5 x 4 / 7	10 x 3 / 4.85		5x 4/ 7	10x3 / 4.85
Spinal Cord + 1 mm	23.75	35	0.1 cm^3^		
Cauda Equina	25	37.5	0.1 cm^3^		
Kidney	-	-		10	12
Bowel	24	37	1 cm^3^	-	-
Esophagus	30	40	1 cm^3^	-	-
Liver		-		12.5	17.5

Normal tissue constraints depending upon the fractionation schema.

#### Treatment delivery

Volumetric cone-beam CT (CBCT) imaging will be obtained at each treatment after patient positioning to verify patient set-up. Another verification scan will be acquired following on-line correction to ensure a residual error of ≤ 1 mm and ≤ 1°, and one following beam delivery to assess for intra-fraction drift.

#### Trial duration and follow-up assessments

The primary endpoint will be pain control assessed 3 months after radiotherapy. Detailed follow-up is outlined in Table 2. Patient follow-up will continue for five years to evaluate the secondary end points of the trial. Patient accrual is estimated to be finished within 2 years.

**Table 2:** **Patient follow**-**up and assessment scheme**

**Assessment**	**Prior RT**	**weekly during RT**	**Post RT**	**1**-**4 weeks**	**6 weeks**	**3 mon**	**6 mon**	**9 mon**	**12 mon**	**18 mon**	**24 mon**
**Physical Examination**	X	X	X		X	X	X		X		X
**Karnofsky performance index**	X	X	X		X	X	X		X		X
**Documentation of Pain** &**Analgesic Use**	X	X	X	Self assessment	X	X	X	Tel.	X	Tel.	X
**EQ**-**5D**	X	X	X		X	X	X		X		X
**QLQ**-**BM22**	X	X	X		X	X	X		X		X
**NCI CTCAE v4 toxicity**		X	X		X	X	X		X		X
**MRI** / **CT imaging**	X				X	X	X		X		X

Tel: patients will be interrogated by a study nurse via telephone.

Self-assessment: patients will be given letters for self-assessment of pain and analgetic use for the four weeks following radiosurgery.

For evaluation of pain a 10 point visual analogue scale will be used [15] and analgesic use will be recorded. Pain response is defined as a reduction of ≥ 2 points at the treated site without analgesic increase. Complete pain response is defined as a pain score of 0 at the treated site with no concomitant increase in analgesic intake. Stable pain is defined as unchanged score or within 2 points of the baseline with no increase in analgesia.

Quality of life will be analysed using the EQ-5D and EORTC QLQ-BM22 questionnaires.

Local control will be defined by repeat imaging (preferably MRT or CT). Progression events are defined as radiological documented disease progression using RECIST-Criteria (Revised Guidelines, Version 1.1, 2009) [30].

Kaplan Meier curves for local tumor control, cancer specific survival and overall survival will be calculated starting from the first day of treatment.

This study will use the International Common Terminology Criteria for Adverse Events (CTCAE) version 4.0 for toxicity and adverse event reporting.

#### Criteria for consideration of study termination

The trial may be stopped prior to meeting the accrual goal if the toxicity of hypo-fractionated spinal radiosurgery is determined to be unacceptable. Acute or late toxicity will be considered unacceptable if the rate of CTCAE v4.0 grade 3 toxicity is greater than 30%, grade 4 toxicity greater than 20%, or any grade 5 toxicity attributable to therapy occurs. Interim analyses of toxicity will be planned after the first 10 patients have been accrued, after an additional 20 patients, and after the total number of evaluable patients have been accrued to the study with adequate follow-up (generally > 90 days after completion of treatment) for assessment of toxicity.

#### Sample size calculation

This study aims to demonstrate an improvement in pain control at 3 months from approximately 40-50% with conventionally fractionated radiotherapy to 70-80% with image-guided, hypo-fractionated radiotherapy. Assuming an improvement in pain control from 45% to 75% sample size calculations at a 5% statistical significant level show that 27 patients would be required to achieve power of the study of 90%. This is the total number of patients eligible for statistical analysis per study arm, i.e. 54 in total. Considering a 10% drop-out rate, the total number of patients is 60. No comparison will be made between the two study arms.

#### Ethical and legal considerations

The DOSIS study is conducted in line with either the Declaration of Helsinki (Tokyo, Venice, Hong Kong, Somerset West and Edinburgh amendments) or the laws and regulations of the country, whichever provides the greatest protection of the patient. The protocol has been written, and the study will be conducted according to the ICH Harmonized Tripartite Guideline for Good Clinical Practice ( http://www.ifpma.org/pdfifpma/e6.pdf).

The trial protocol, patient information and informed consent sheets have been approved by the independent ethics committee of the University Hospital, University of Wuerzburg and the ethics committees of the Royal Marsden Hospital NHS Foundation Trust.

The study protocol has been reviewed by the “Independent Expert Committee of the DEGRO” and was considered as application of therapeutic radiotherapy within the frame of current health care.

#### Sponsorship

For patients treated at the Royal Marsden Hospital, the study sponsor is the Royal Marsden NHS Foundation Trust, Downs Road, Sutton, Surrey, SM2 5PT, United Kingdom. For patients treated at the Department of Radiation Oncology of the University Hospital Wuerzburg, the study sponsor is the University Hospital Wuerzburg, Wuerzburg, Germany.

## Discussion

This study will assess efficacy and safety of dose-intensified radiosurgery for painful vertebral metastases. Data from this study will help to model a potential relationship between irradiation dose and pain palliation. Such a dose–response relationship has not been demonstrated in randomized trials before: we hypothesize that irradiation doses in all arms of the randomized trials were in the flat region of the sigmoid-shaped dose–response curve and higher doses are required to improve clinical results. The modern technologies of IMRT and IGRT, which are mandatory in this study, will be used to realize planning and delivery of such escalated doses safely. Despite pain control at three months being the primary endpoint, patients will remain in follow-up for five years for analysis of long-term pain control, local tumor control and toxicity.

Follow-up was limited in the majority of the existing studies due to short overall survival of unselected patients. Patients with estimated short life expectancy will be excluded from this study, which will allow evaluation of long-term efficacy and in particular safety of intensified radiosurgery in this palliative setting. More reliable data on long-term safety with a particular focus on radiation induced myelopathy is considered highly important for further development of spine radiosurgery.

Fractionated radiosurgery allows the inclusion of patients, whose tumor actually contacts the spinal cord. Fractionation will enable higher biological doses at the target / organ-at-risk interface. This is expected to reduce the local failures, which were most likely the result of underdosing the volumes in this high-risk region. Patients with the metastases approaching the spinal cord but without neurological deficits are not typical surgical candidates and local radiotherapy with durable tumor control might be especially warranted in this patient collective for prevention of MSCC.

Fractionation might also be beneficial from a safety perspective. Despite inter- and intra-fractional patient motion being minimized by IGRT and patient immobilization devices, residual errors need to be considered and cannot fully be avoided. Fractionation is expected to limit the consequences of such errors unless they are systematic and occur on a daily basis. This will be avoided by strict quality assurance.

In conventional radiotherapy for spinal metastases, the involved vertebral levels as well as the two adjacent vertebrae were defined as target volume. This was practiced because of uncertainties of disease extension as well as low accuracy of treatment delivery. We will limit the target volume to the involved vertebrae based on radiosurgical experiences of low recurrence rates in the adjacent non-involved vertebrae [17,25,31], but regional control in these vertebrae will be a secondary endpoint of this analysis. In contrast to most radiosurgical studies, we will continue treating the whole metastatic vertebrae. This will be achieved using the simultaneous integrated boost approach, where dose intensification will be applied to the involved parts of the vertebrae only and uninvolved parts will be treated with conventional doses. It is intended to perform a detailed analysis of recurrences with dose effect modelling, which will help to refine evidence-based target volumes for future trials.

It is the intention of this study to form the basis for a future phase III trial comparing conventional radiotherapy with radiosurgery for palliation of painful vertebral metastases in a randomized manner. The methods of patient selection, target volume definition, treatment planning and delivery as well as quality assurance will be redefined based on the results of this study.

## Competing interests

The authors declare that they have no competing interests.

## Authors’ contributions

MG and RAS designed the study and MH and MF participated in the design of the study. All authors performed critical review of the manuscript and finally approved the manuscript.

## Pre-publication history

The pre-publication history for this paper can be accessed here:

http://www.biomedcentral.com/1471-2407/12/530/prepub
